# Evaluation of Stress in the Maxillary Complex of a Unilateral Cleft Lip and Palate on Simulated Occlusal Loading Using Finite Element Analysis

**DOI:** 10.1007/s12663-025-02458-8

**Published:** 2025-02-12

**Authors:** Fredrick Williams, Afiya Eram, Mohammad Zuber, G. Srikanth, Anupam Singh, Rajendra Prasad, S. M. Abdul Khader, A. B. V. Barboza

**Affiliations:** 1 Department of Oral and Maxillofacial Surgery, A.B. Shetty Memorial Institute of Dental Sciences, Mangalore, 575018 India; 2 Department of Conservative Dentistry and Endodontics, Manipal College of Dental Sciences, Manipal Academy of Higher Education, Manipal, 576104 India; 3 Department of Aeronautical and Automobile Engineering, Manipal Institute of Technology, Manipal Academy of Higher Education, Manipal, 576104 India; 4 Department of Oral and Maxillofacial Surgery, Manipal College of Dental Sciences, Manipal Academy of Higher Education, Manipal, 576104 India; 5 Department of Mechanical and Industrial Engineering, Manipal Institute of Technology, Manipal Academy of Higher Education, Manipal, 576104 India

**Keywords:** Finite element analysis, Unilateral cleft lip and palate, Maxillary complex, Stress and displacement, Simulated occlusal loading

## Abstract

**Background:**

In individuals with a unilateral cleft lip and palate, there routinely exists an abnormality of the facial skeleton in all three planes **(**transverse, sagittal and coronal**)**. Skeletal and facial asymmetry is pronounced in the anterior part of the maxilla with a smaller maxillary width and height on the cleft side. As a mechanical stimulant, occlusal forces and the resulting stress and strain distribution within the skeletal components lead to strain-induced bone remodeling. This study was done to observe the stress distribution pattern and displacement within the maxillary complex in a complete unilateral cleft lip and palate individual when subjected to simulated occlusal forces, using a three-dimensional finite element analysis.

**Material and Methods:**

A three-dimensional finite element model of the maxillary complex of a unilateral cleft lip and palate individual was developed from sequential computed tomography scan images processed at 1-mm intervals. ANSYS™ 14.0 and MIMICS™ software were used for the same. Masseter forces of 300 N were applied at the zygomatic arch bilaterally, and occlusal loads of 100 N were applied vertically onto the framework surface at different locations to simulate occlusal loading. The displacement and von Mises stresses in different planes were studied on different nodes at various anatomical points within the maxillary complex.

**Results:**

The unilateral cleft lip and palate led to a non-uniform, asymmetric stress distribution pattern within the maxillary complex: intensified on the non-cleft side and weakened on the cleft side. An asymmetric displacement pattern was noted between the cleft and non-cleft sides.

**Conclusions:**

The results implied that an individual born with a complete unilateral cleft lip and palate would be expected to have an asymmetric facial development between the non-cleft and cleft sides as a result of an asymmetric occlusal loading pattern.

## Introduction

Clefts of the lip and palate are the most common craniofacial birth defect and the second most common congenital abnormality, with a birth prevalence ranging from 1 in 500 to 1 in 2000, depending on the population [1]. The affected child may undergo multiple-staged reconstructive procedures to restore the underlying maxillofacial skeleton and the overlying soft tissue. Individuals with a unilateral cleft lip and palate manifest a plethora of phenotypic characteristics, including abnormality of the facial skeleton in all three planes (transverse, sagittal and coronal). Skeletal asymmetry is pronounced in the anterior part of the maxilla with a narrower maxillary width and height on the cleft side [2]. A reduction in the vertical development of the maxilla on the cleft side as well as a collapse of the arch segments in the transverse plane is usually noted in these individuals [1, 3]. Striking asymmetries of the soft tissues as well as of the nasomaxillary and lower facial structures are noted. Clinically, it is observed that such skeletal asymmetry appears minor in infancy and becomes severe in adolescence.

Facial skeletal growth in a child born with a facial cleft is regulated by both intrinsic and functional factors such as genetics, soft tissue matrix, mechanical stimulation and iatrogenic causes [1]. Among these, functional loading, specifically the occlusal forces acting on the dentofacial skeleton, play a significant role. As a mechanical stimulant, these occlusal forces and corresponding stress and strain distribution fields within the maxillary complex, lead to strain-induced bone remodeling [1]. It is therefore conceivable that the interaction between the occlusal forces in a complete unilateral cleft lip and palate individual, would lead to an asymmetrical stress and strain distribution in the maxillary complex, resulting in an asymmetrical growth and development.

The biomechanical functions of teeth generally result in stresses, which are transferred from the teeth to the maxillofacial complex, and produce zones of stress and strain. Understanding the nature of strain and stress distribution is essential for better diagnosis and treatment of stomatognathic diseases and reconstruction of masticatory function. Unfortunately, the stresses cannot be measured directly in a non-destructive way. The number of direct studies on the effects of the masticatory system on the maxillary complex in the cleft individual is limited, because its structures are difficult to reach and the applications of experimental devices, such as strain gauges, inside the structure cause damage to its tissues, which could interfere with normal function and influence their mechanical behavior [4]. Lately, the advent of the finite element (FE) method proved to become a powerful analysis instrument of structural behavior and interaction analysis of bodies, systems and environments of a different nature. A remarkable advantage of the finite element method is the chance to study areas that are difficult or impossible to access without any risks to a living subject of investigation [4].

The use of finite element method in this study allows a more precise biomechanical basis for analysis when compared to other methods such as photoelastic models and strain gauges [4]. Development of a 3-D finite element model of the human maxillary complex has been difficult because of the anatomic complexity, hollow structures and large variation in topography and bone thickness. This study intended on developing a finite model that accurately simulates the forces acting on the maxillary complex in a unilateral cleft lip and palate individual on simulated occlusal loading.

## Methodology

**Fig. 1:**
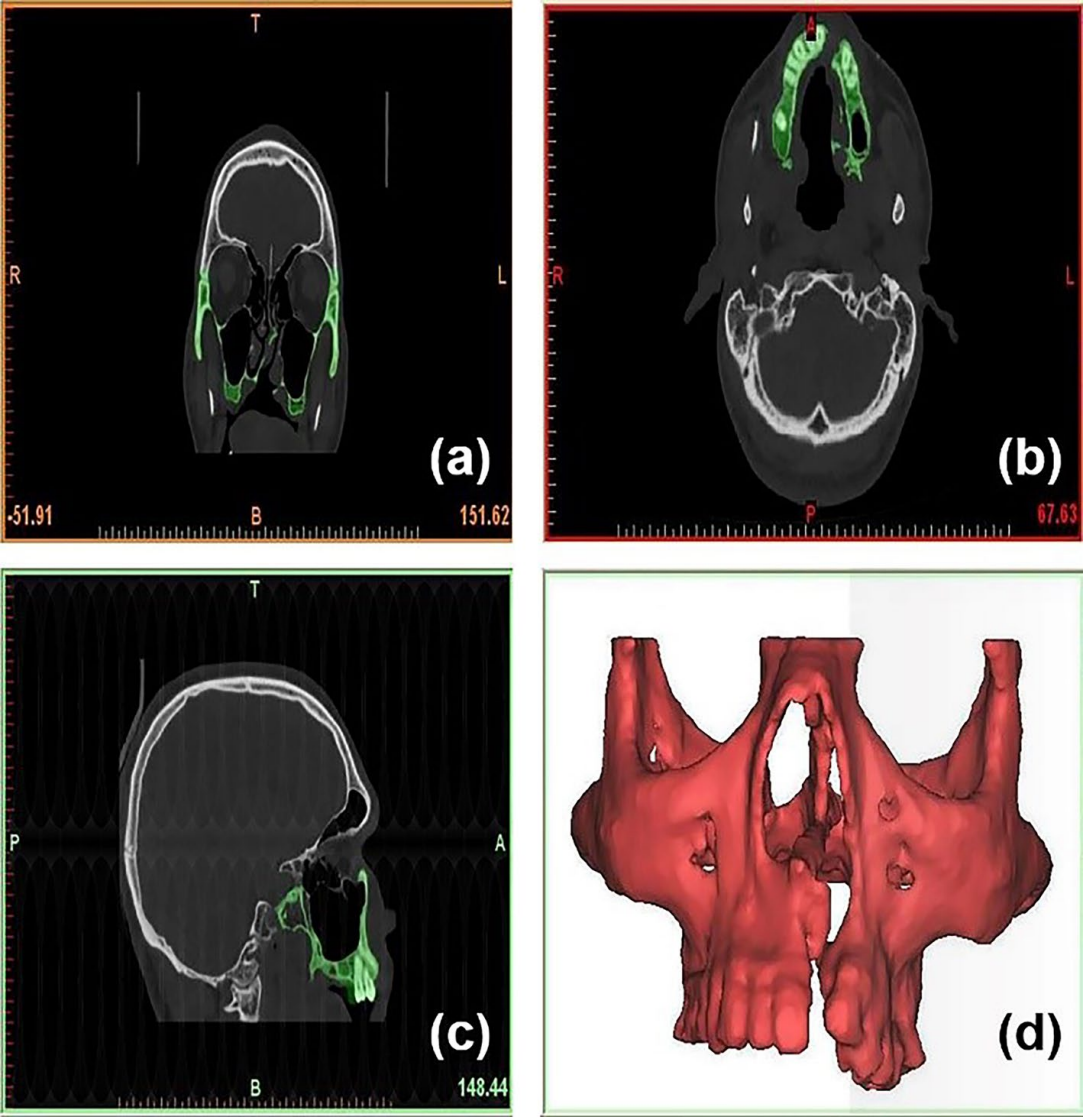
CT scan images from **a** axial, **b** coronal, **c** sagittal plane and **d** 3-D model of maxillary complex of a unilateral cleft lip and palate

The present study was conducted to observe the stress distribution pattern and displacement in the maxillary complex of a complete unilateral cleft lip and palate individual on simulated occlusal loading using 3-D finite element analysis. It was done by constructing a 3-D finite element model of a human maxillary complex using finite element software—ANSYS 14.0, with the aid of CT scan digitization. The model was then assessed by comparing the stress distribution pattern and displacement between the cleft and non-cleft sides. Subject-specific computerized tomography (CT) scan was obtained from a male adolescent with a complete unilateral cleft lip and palate, procured from K.S Hegde Medical Academy, Mangalore. Sequential CT images were processed at 1-mm intervals and had more than 300 slices each in the coronal, sagittal and axial planes as shown in Fig. 1. After the hard tissue was separated from the soft tissue using a proper threshold value, the maxillary complex was separated from the mandible and the skull. The CT scan images were read into visualization software: MIMICS (Materialise Interactive Medical Image Control System). MIMICS is an interactive tool for the visualization and segmentation of CT images and 3-D rendering of objects.

**Fig. 2:**
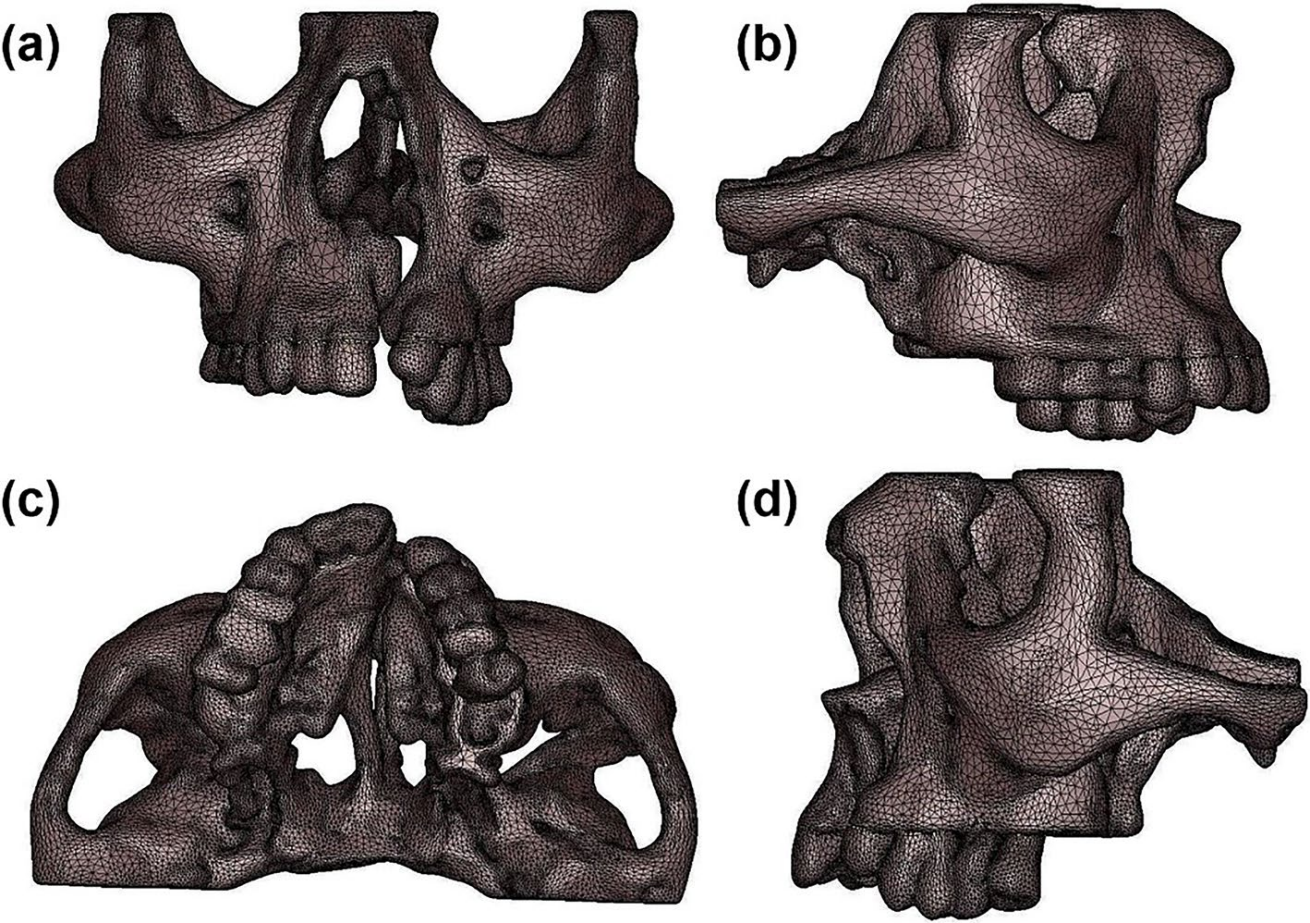
Meshing observed under **a** anterior, **b** right lateral, **c** bottom and **d** left lateral views of maxillary complex of a unilateral cleft lip and palate

After manual refinement of the model (i.e., cleaning, repairing and smoothing), volumetric meshes were generated using tetrahedral elements. The model consisted of 1,45,000 tetrahedral elements and 34,000 nodes representing teeth and 3,41,900 elements and 96,200 nodes representing both cortical and cancellous bone with an edge length of 0.5. The details of the mesh are shown in Fig. 2. A fine mesh ensured that the errors in the calculated stress and strain components would be less than 3.0%0.1. The complete geometry was now defined as an assemblage of discrete pieces called elements, which are connected to each other at a finite number of points called nodes. In this study, 3-D 10-noded tetrahedral solid elements commercially named as SOLID92 were used as an element type. SOLID92 has a quadratic displacement behavior and is suited to model irregular solid geometry, and gives a better representation of the variable bone thickness that is characteristic of the maxillofacial skeleton. The element was defined by ten nodes having three degrees of freedom at each node: translations in the nodal x, y and z directions.

**Fig. 3:**
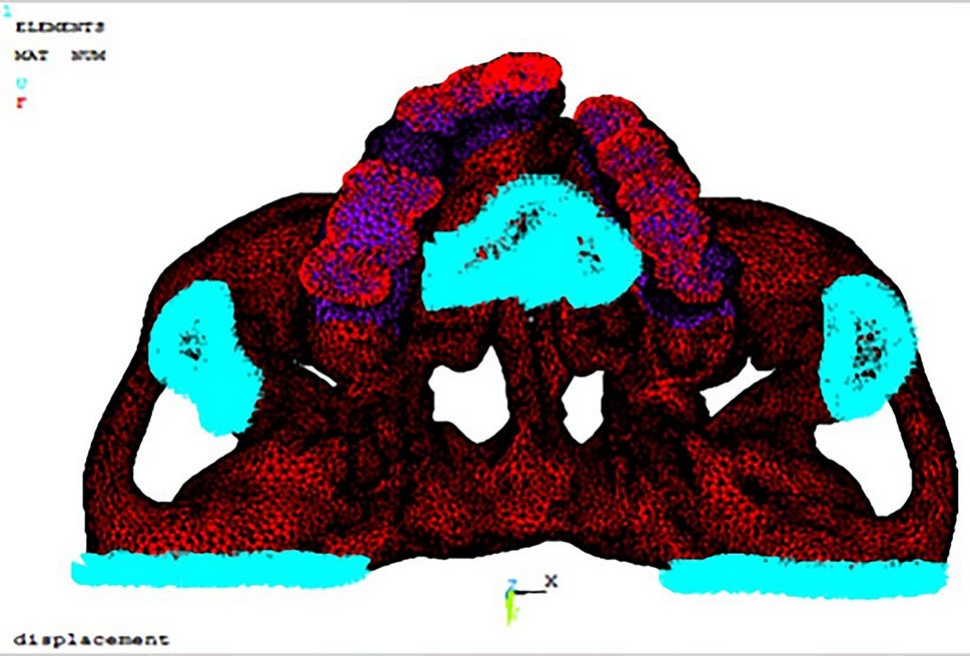
Boundary conditions assigned for the model

The materials in the analysis were assumed to be linearly elastic and isotropic. For the FE model, the material properties were assigned to the cortical bone, cancellous bone and teeth based on the Hounsfield units. Assumptions were made similar to other FE studies for the material characteristics of bone [5]. Cortical bone is isotropic, homogeneous and linearly elastic with a thickness of 1.5 mm [6]. The boundary conditions in the FE model basically represent the load imposed on the structures under the study and their fixation counter parts. The points at the frontal process and the zygomatic process were constrained to have no motion in any direction. For the FE model, the inferior and posterior ends of the maxilla were fixed without movement, while occlusal loads (nodal forces) were added to the teeth perpendicular to the occlusal plane, creating a total force of 100 N. The simulated occlusal loading was applied as a vertical load at different locations on the framework surface, specifically at the central incisor (L1), first premolar (L2), first molar (L3) and second molar region (L4) on the non-cleft side and the canine (L1), first premolar (L2), first molar (L3) and second molar region (L4) on the cleft side to represent more realistic food positions during biting or chewing [7]. These directions and points of application were selected to simulate physiological patterns of contact during occlusal loading/chewing (intercuspal position and laterotrusive and mediotrusive movements). A maximum load of 100 N is within physiological limits during chewing [8]. All vertical loads were applied along the z- axis. Furthermore, a distributed force of 300 N, with force components of − 12.42 N along the x-axis, -53.04 N along the y-axis and 25.14 N along the z-axis were applied to the insertion area of the masseter muscle on the zygomatic arch to simulate action of this muscle [7, 9]. The boundary conditions assigned are shown in Fig. 3 and Tables 1 and 2.

**Table 1:** Material properties of the bones in the maxillary complex

Material	Young’s modulus	Poisson’s ratio Poisson’s ratio
Cortical bone	13.4 × 10^3^	0.3
Cancellous bone	77 × 10^2^	0.3
Teeth	19.6 × 10^3^	0.3

**Table 2:** Computational results of the transverse (X), sagittal (Y) and vertical (Z) displacement of various skeletal structures of the maxillary complex on simulated occlusal loading on the non-cleft side and cleft side, respectively:

Region	Selected Nodes on	Non-cleft side	Cleft side				
		X (mm) Poisson’s ratio	Y (mm)	Z (mm)	X (mm) Poisson’s ratio	Y (mm)	Z (mm)
Dentoalveolar	Incisal edge of central incisor	0.0016	0.0012	0.0015	–	–	–
	Cuspal tip of canine	0.0014	0.0065	− 0.0062	− 0.038	0.0072	− 0.0007
	Cuspal tip of 1st permanent molar	0.018	0.0075	− 0.0064	− 0.042	0.0081	− 0.0035
	Apical region of central incisor	0.0015	0.0023	0.0018	–	–	–
	Apical region of canine	0.0014	0.004	− 0.0072	− 0.035	0.0052	− 0.0032
	Apical region of 1st permanent molar	0.0175	0.0046	− 0.0064	− 0.042	0.0078	− 0.0038
Maxilla	Point A	0.0015	0.0031	− 0.0032	− 0.029	0.0065	− 0.0042
	Body	0.0016	0.0055	− 0.0062	− 0.025	0.0032	− 0.0065
	Tuberosity	0.018	0.0048	− 0.0072	− 0.026	0.0086	− 0.0008
	Zygomatic buttress	0.016	0.0091	− 0.0015	− 0.0195	0.0053	− 0.0017
	Inferior orbital rim	0.0017	0.0013	− 0.0093	− 0.011	− 0.002	− 0.015
	Frontal process	0.0018	− 0.0035	− 0.0012	− 0.0095	− 0.0028	− 0.0093
Palate	Anterior	0.00165	0.0098	− 0.0035	− 0.025	− 0.0073	0.0038
	Posterior	0.0019	0.0065	0.0016	− 0.027	− 0.01	0.0095
Zygomatic bone	Frontal process	0.0018	− 0.0042	− 0.0074	− 0.0076	0.0016	− 0.0094
	Temporal	− 0.0085	− 0.0028	− 0.0035	0.0013	0.0013	− 0.0039
	Maxillary	0.018	0.0072	− 0.0098	− 0.015	0.0055	− 0.0142
	Body	0.0016	0.0014	− 0.0165	− 0.0065	0.0085	− 0.0155
Nasal cavity wall	Nasal septum	− 0.0095	− 0.0042	0.0018	0.0011	− 0.0013	− 0.0021
	Anterior nasal spine	0.00151	0.0094	− 0.0032	0.0015	0.0036	− 0.0008
	Nasal bone	− 0.0092	− 0.0052	− 0.0008	0.0014	− 0.0024	− 0.0009
	Antero-inferior	0.00168	0.0018	− 0.0035	− 0.018	0.0024	− 0.0032
	Antero-superior	− 0.0092	− 0.0035	− 0.0042	− 0.0063	− 0.002	− 0.0041
	Postero-inferior	− 0.0086	0.0024	0.0065	− 0.024	0.0036	0.0065
	Postero-superior	0.0012	− 0.0042	− 0.0043	0.0011	0.0019	0.0042

## Results

**Fig. 4:**
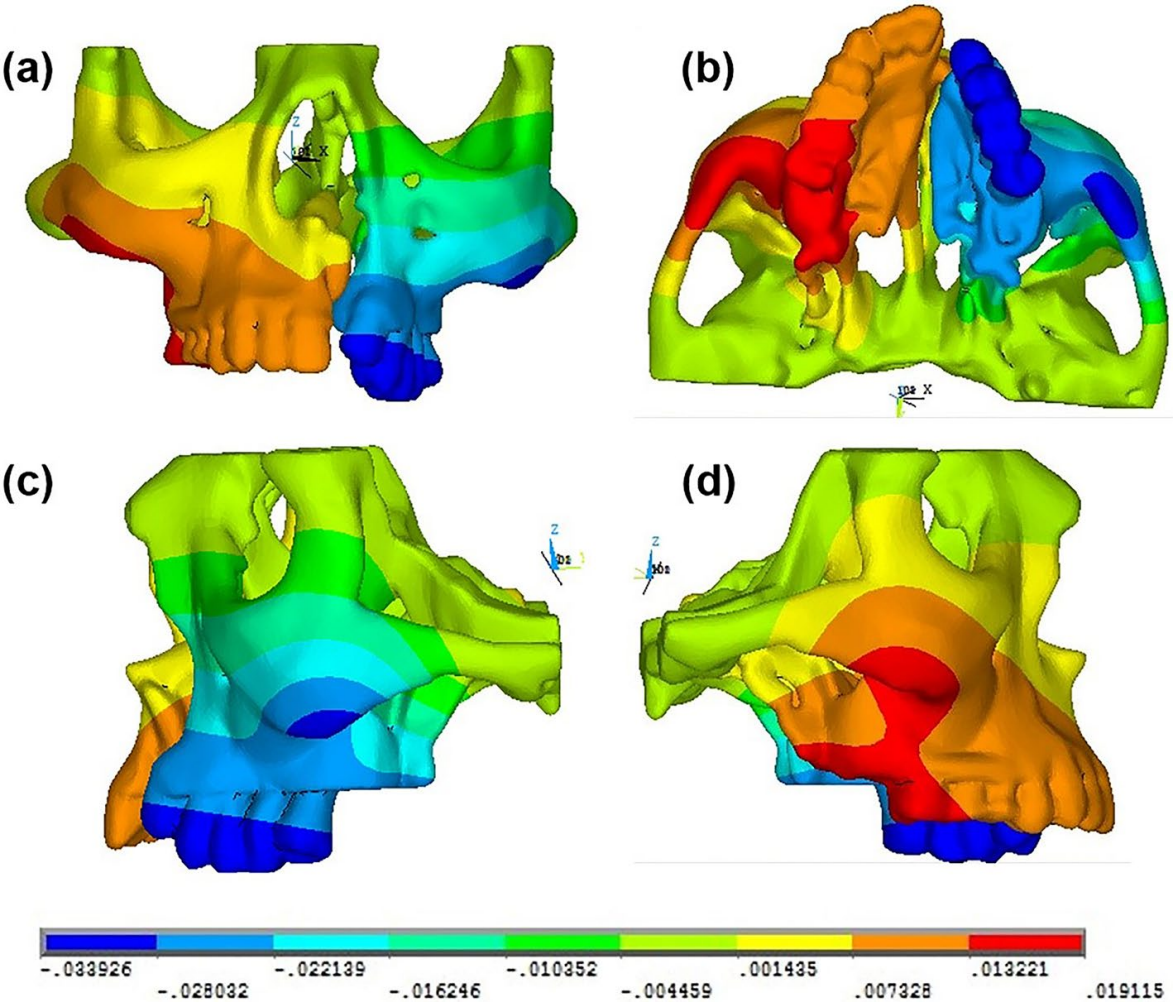
Pattern of computed transverse (X) displacement in **a** anterior, **b** bottom, **c** left lateral and **d** right lateral views of the skeletal structures in the maxillary complex

**Fig. 5:**
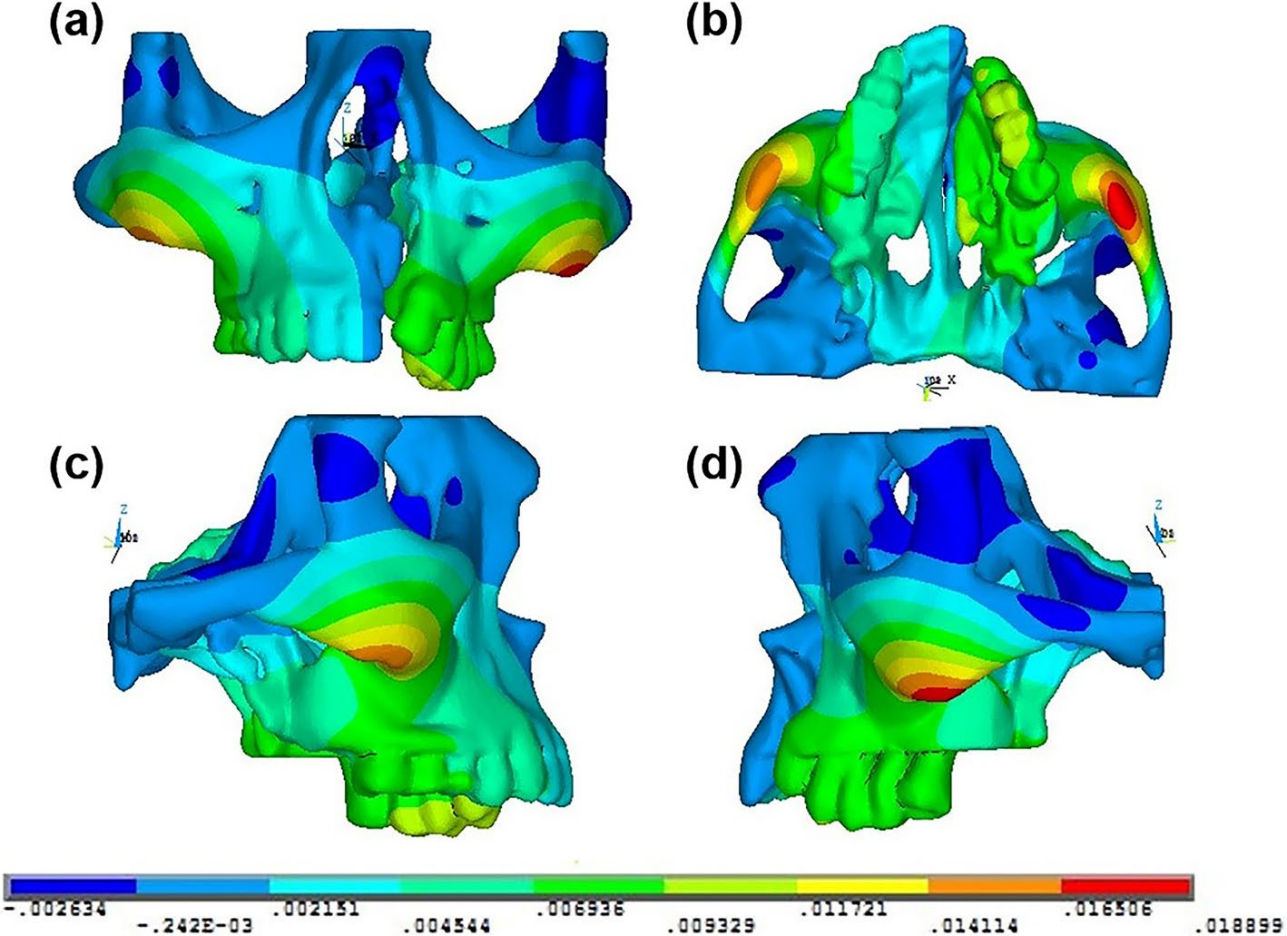
Pattern of computed sagittal (Y) displacement in **a** anterior, **b** bottom, **c** right lateral and **d** left lateral views of the skeletal structures in the maxillary complex

**Fig. 6:**
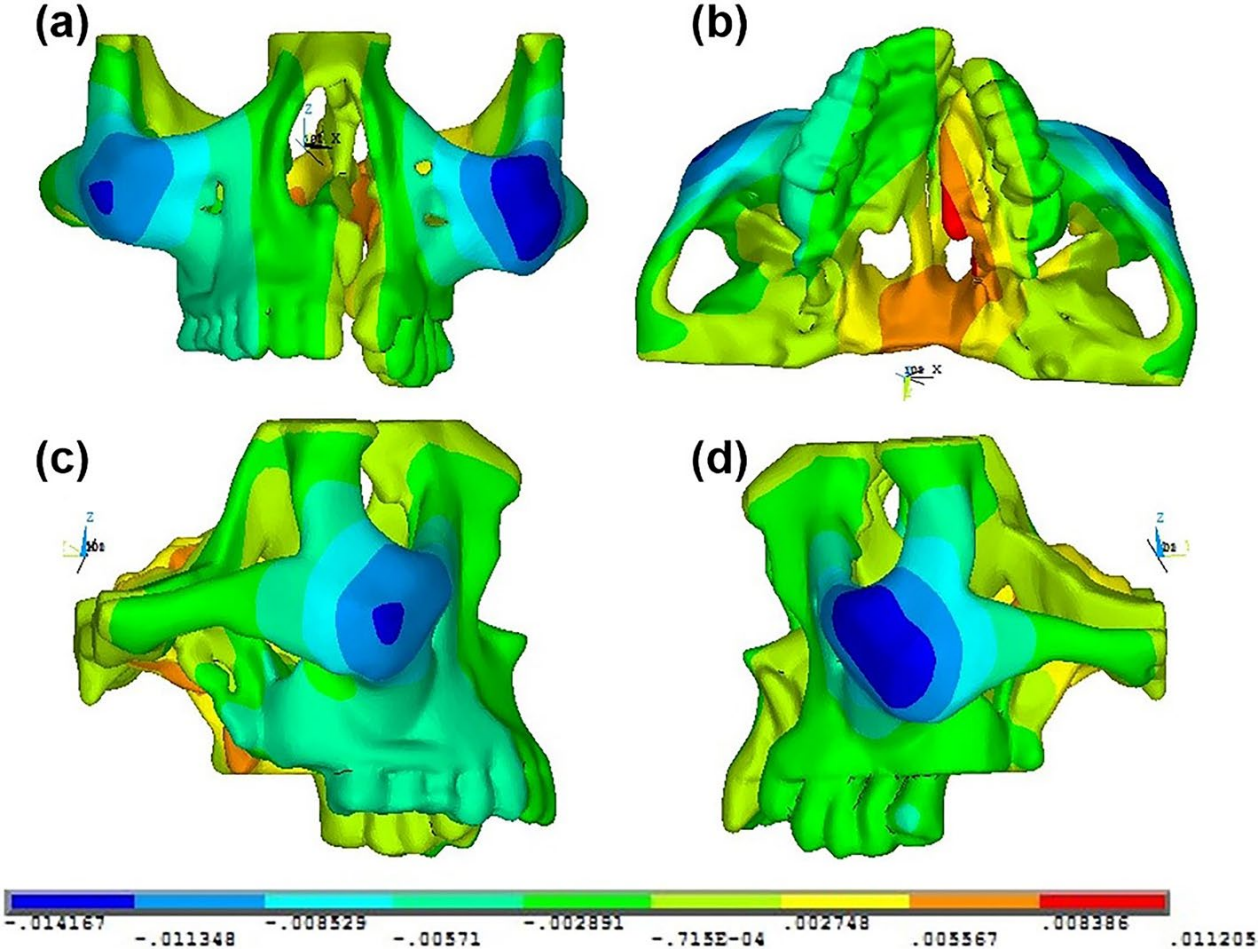
Pattern of computed vertical (Z) displacement in **a** anterior, **b** bottom, **c** right lateral and **d** left lateral views of the skeletal structures in the maxillary complex

The simulations were then carried out, and the von Mises stresses and displacement at various anatomical points within the maxillary complex of the complete unilateral cleft lip and palate patient were analyzed; the results were represented diagrammatically with color codes and tabulated. The values of the strain are indicated by different color contours, and the positive and negative signs represent the state in tension and in compression, respectively. To interpret the displacements in the images of transverse direction (X), the (+ ve) value is represented by yellow, orange and red and displacement in the opposite side is represented by a blue color, as shown in Fig. 4. For displacements in the sagittal direction (Y), the (+ ve) value is represented by shades of blue, green, yellow, orange and red respective to the corresponding values which indicates an ‘anterior movement’. The (− ve) value is represented by darker shades of blue, which corresponds to a ‘posterior movement’, as shown in Fig. 5. In the images of displacement along the vertical direction (Z), the (+ ve) value is represented by yellow, orange and red which indicates an ‘upward movement’. The (− ve) value is represented by shades of green and blue, indicating a ‘downward movement’, as shown in Fig. 6.

**Fig. 7:**
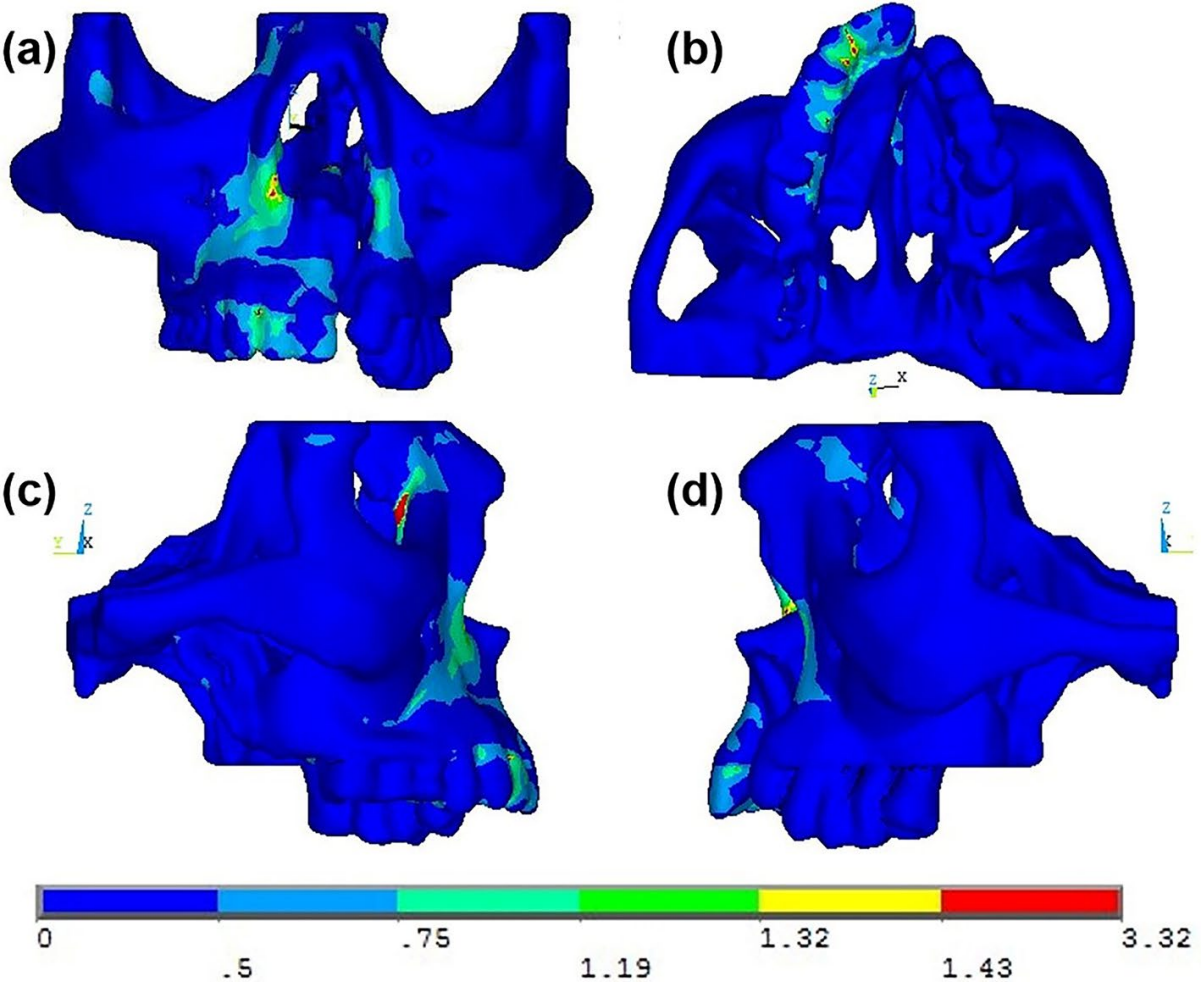
Pattern of stress distribution in the maxillary complex on application of occlusal forces seen in **a** anterior, **b** bottom, **c** right lateral and **d** left lateral views

**Fig. 8:**
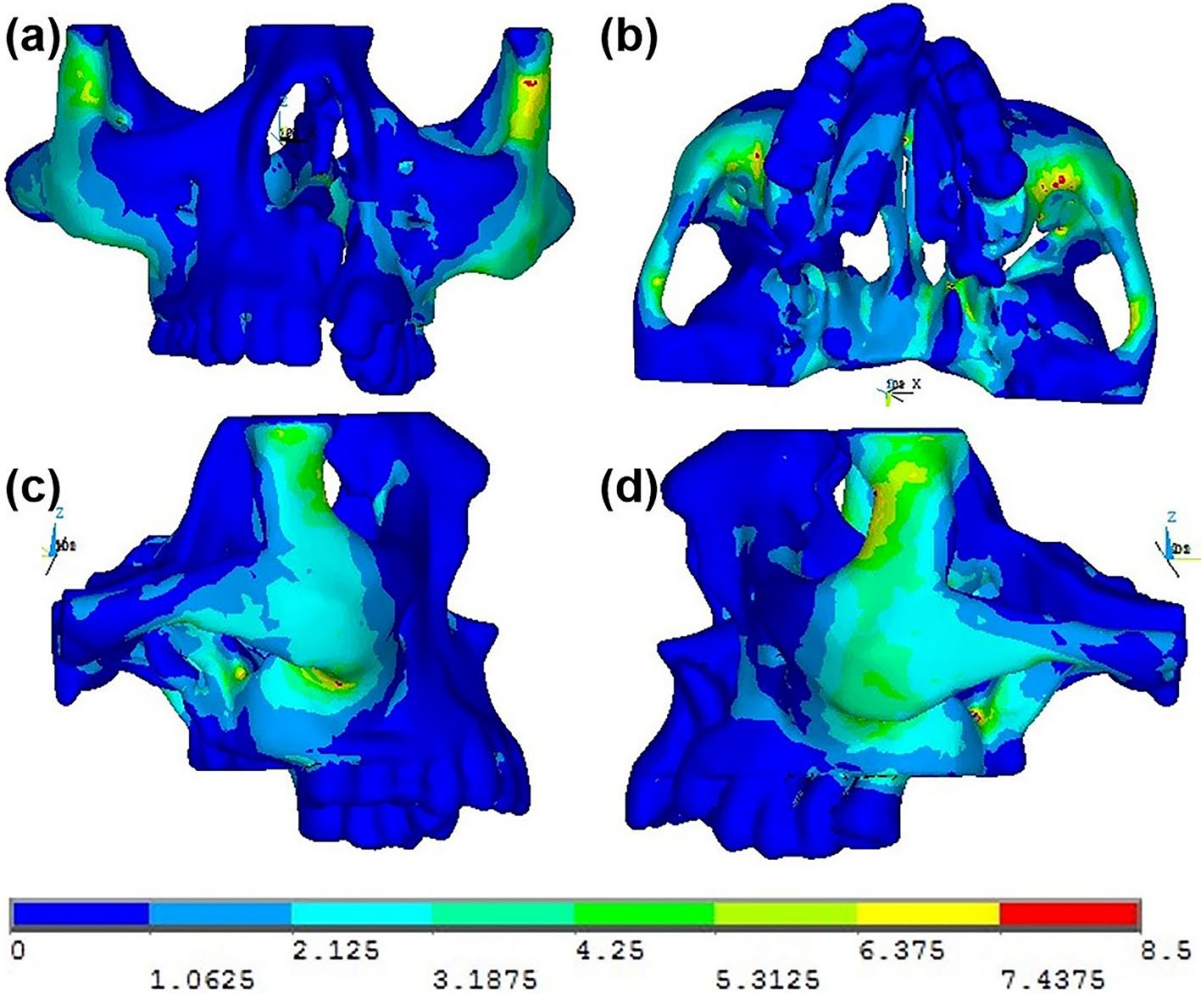
Pattern of stress distribution in the maxillary complex due to the action of masseter muscle seen in **a** anterior, **b** bottom, **c** right lateral and **d** left lateral views

Magnitude and distribution of maximum von Mises stresses produced at various areas of the maxillary complex of a unilateral cleft lip and palate on simulated occlusal loading on both the non-cleft and cleft sides are shown in Table 3. The images depicted in Fig. 7 represent the von Mises stress distribution in the maxillary complex on simulated occlusal loading. The images in Fig. 8 represent the von Mises stress distribution in the maxillary complex on simulated occlusal loading showing the stress distribution during the action of the masseter muscle.

**Table 3:** Von Mises stresses seen at various structures in the maxillary complex on the non-cleft and cleft sides on simulated occlusal loading

Region	Selected nodes on	von Mises stress (N/mm^2^)	
		Non-cleft side	Cleft side
Palate	Incisal edge of central incisor	0.62	0.21
	Cuspal tip of canine	0.51	0.23
Maxilla	Point A	0.65	0.34
	Body	0.45	0.22
	Tuberosity	0.34	0.22
	Zygomatic buttress	0.35	0.25
	Inferior orbital rim	0.43	0.34
	Frontal process	0.71	0.41
Zygomatic bone	Frontal process	5.76	7.35
	Temporal	1.85	1.65
	Maxillary	2.45	3.82
	Body	2.65	2.45
Nasal cavity wall	Nasal septum	3.15	0.25
	Anterior nasal spine	3.34	0.14
	Nasal bone	0.61	0.31
	Antero-inferior	5.85	0.53
	Antero-superior	0.34	0.22
	Postero-inferior	0.22	0.65
	Postero-superior	1.17	0.91

## Discussion

Growth and development of the maxillofacial complex is of tremendous interest to scientists, clinicians, and even the general public. Most craniofacial anomalies and dentofacial deformities result from inherited mutations and aberrant environmental modulation of multiple genes. Mechanical forces readily modulate the bone and cartilage growth [10]. An exogenous force must possess certain characteristics before it qualifies as a mechanical stimulus, defined as a mechanical signal capable of eliciting anabolic or catabolic growth response. The three-dimensional finite element analysis applied in the mechanical analysis of stresses and strain in the field of engineering makes it possible to elucidate the biomechanical state variables such as displacement, strain and stress induced in living structures by various external forces.

Patients with clefts of the lip and palate present with an abnormal skeletal structure and facial morphology as a result of intrinsic, functional and iatrogenic causes [10]. In patients with a unilateral cleft lip and palate, asymmetric development of the facial skeleton in all three planes (transverse, sagittal and coronal) has been documented in literature [11]. A multitude of variables can influence the outcome leading to such asymmetry, such as the geometry of the cleft itself and the resulting differences in the transfer of occlusal loading forces that can influence subsequent upper facial skeletal development and remodeling [1]. The complete unilateral cleft lip and palate introduces an asymmetry in the facial skeleton and the extent to which it contributes to the skeletal deformity remains to be elucidated.

The finite element methods previously had been used to model the load transfers within the skull and the maxilla [12]. However, none of these studies addressed the clinically relevant situation in which the facial skeleton is disrupted by a cleft. As the first step toward developing a clinically relevant, patient-specific FEM, the present study focused solely on the biomechanical response to the occlusal forces in the maxillary complex of a unilateral cleft lip and palate. During masticatory function such as incising, masticating and chewing, the functional loads are transmitted through the dentition to the maxilla and upper mid-facial skeleton. In an unaffected maxilla, the transmission of occlusal loads would be expected to result in symmetrical stress and strain distribution within the mid-facial skeleton to the extent of the symmetry of the facial skeleton [1].

The current FEM model revealed stress concentration zones at the medial nasomaxillary buttresses on both the non-cleft and cleft sides, with a higher stress concentration observed on the non-cleft side as well as at the lateral zygomatic-maxillary buttresses. These locations clinically correspond to the structural buttresses where the bone is thicker anatomically [1]. A wider area of stress distribution was also observed on the non-cleft side of the maxillary complex when compared to the cleft side. The presence of a unilateral cleft lip and palate breaches the structural integrity of the skeleton and alters the transmission pattern of the functional loads within the maxilla, which is transmitted primarily through the non-cleft side rather than the cleft side. Correspondingly, the stress and strain distribution on the maxillary complex with a complete unilateral cleft lip and palate is both non-uniform and asymmetric with regard to the mid-sagittal plane, i.e., the stresses and strain intensified on the non-cleft side and decreased on the cleft side. These predictions confirm our first hypothesis that the complete unilateral cleft lip and palate leads to a non-uniform and asymmetric stress and strain distribution pattern in the maxillary complex of such an individual. Such an asymmetric stress and strain distribution would suggest an asymmetrical skeletal development on the non-cleft side when compared to the cleft side.

The fact that a certain level of stress and strain, corresponding to the threshold strain, is required to trigger the strain-induced bone modeling [13], the skeletal segments with higher stress and strain levels, such as the non-cleft side, would be expected to have a greater potential to develop and grow in response. In contrast, the segment with lower stress and strain levels, such as the cleft side, is less likely to further develop and grow. This may provide a possible explanation, from a biomechanical point of view, as to why the individual with a unilateral cleft lip and palate develops a differential between the two maxillary segments both in volumetric size and in spatial dimensions [3]. However, our study does not exclude the effect of other extrinsic, intrinsic and iatrogenic factors that may contribute to the mid-facial skeletal asymmetry.

As a preliminary study on a patient-specific cleft lip and palate, certain assumptions were necessary to simplify the model and to emphasize the stresses in the maxillary complex. The application of a linear elastic material model to the maxilla neglected the nonlinear dynamic responses of the bony materials. The homogeneous and isotropic assumption simplified the structure of the maxillary bone and especially the unique load-bearing feature of the dentition and the surrounding soft tissues such as the periodontal ligament. Additionally, assumptions about material properties of the dentition and periodontal tissues, together with the simulated occlusal loading forces at the dentition, would lead to a less accurate prediction within the immediate dental region. Whereas the material model was more accurate with regard to the maxillary complex as a whole, the loading effect, according to St. Venant’s principle, would be less likely to significantly influence the predictions in the region of interest that are away from where the loads were applied. Therefore, the stress and strain pattern on the maxillary complex, away from the dentition, would be expected to be more accurate.

Previous studies on the facial skeletal development revealed that individuals with unilateral cleft lip and palate present with a significant degree of facial asymmetry as seen on frontal cephalometric radiographs at the level of the nasomaxillary complex [13], as well as of the lower facial skeleton [14]. When the stress patterns were evaluated, the results of this study were similar to the studies of Zhao et al., who also concluded that the stresses were intensified on the non-cleft side [1]. The stress distributions in these studies were accepted as uneven and asymmetric with regard to the midline plane. In the said study, a clefting pattern was introduced to a normal maxilla in a FE model to study the stress and strain distribution. However, material properties to separate cancellous, cortical bone and teeth were not included. The action of the masseter muscle during occlusal loading was also not taken into consideration. The fact that individuals with unilateral cleft lip and palate display a unilateral malformation allowed us to utilize the measurements of the contralateral non-cleft side of the individual as internal control. However, the fact that normal and ‘clefting’ occur in the same individual should be taken into consideration when interpreting the results, since it is possible that the development of the cleft side could influence the control side within the same individual [15].

Biomechanical models of the human maxillary complex and the masticatory system are not perfect, while they are based on a number of assumptions and simplifications. The adequacy of the FE computational model to the real system depends on the correctness of representation of the geometry and material properties of the modeled object, the type and number of elements and the boundary conditions imposed on the model. The point of application, magnitude, and direction of forces may easily be varied to simulate the clinical situation. Thus, FEM would be an effective approach in the investigation of the biomechanical behavior of the maxillofacial skeleton in all three planes. It should also be noted that the structure and spatial relationships of various craniofacial components vary among individuals with cleft lip and palate. These factors may contribute to varied responses of the maxillary complex on simulated occlusal loading. Though the results of the study are valid only for a patient-specific maxillary complex, the displacement can be a baseline for a larger population. Determination of strains and stresses in the maxillary complex of a unilateral cleft lip and palate patient under simulated occlusal loading has an important impact in different clinical situations. From a biological view, it is known that strains determine to a great extent the functional behavior of bone cells. Therefore, knowledge of this parameter may permit assessment of the regenerative capacity of bone turnover in various states (fracture healing, callus stabilization or transplant healing).

Concerning the biomechanics of bones, stress evaluation in various anatomical zones can be used to investigate potential fracture sites under simulated loading. Additionally, new prostheses design for the rehabilitation of cleft palate patients can be improved, through the help of combined assessment of stresses and strains. Hence, it may be possible to minimize facial disfiguration in affected patients by addressing the mid-facial asymmetries in the growing cleft patients at early developmental stages. The maxillofacial surgeon can make use of stress distribution for better treatment for correcting jaw anomalies. The study also helps to estimate patient-specific tooth load distribution in a complete unilateral cleft lip and palate patient. This could provide reference data for studies on the biomechanics of prosthetic devices used in the rehabilitation of cleft patients.

## Conclusion

In the present study, stress analysis on maxillary complex of a unilateral cleft lip and palate on simulated occlusal loading has been investigated using finite element analysis. It has been observed that in the non-cleft side, the maximum stress initially concentrated in the maxillary complex is at the antero-inferior part of the nasal cavity wall. However, on the cleft side, the maximum stress initially concentrated in the maxillary complex is at the frontal process of the zygomatic bone. There existed an asymmetric displacement and stress distribution pattern between the cleft and non-cleft sides, with a wider area of stress distribution observed on the non-cleft side of the maxillary complex. Therefore, an asymmetric stress distribution pattern in the maxillary complex of a complete unilateral cleft lip and palate on simulated occlusal loading would be expected to lead to asymmetric facial development.
